# On the Improved Method of Treatment of Diseases of the Lungs, Throat, and Nasal Passages, by Inhalations of Atomized Fluids

**Published:** 1867-09

**Authors:** Wm. C. Lyman

**Affiliations:** Late Surgeon U. S. Navy, Resident Physician U. S. Marine Hospital, etc.


### ON THE IMPROVED METHOD OF TREATMENT OF DISEASES OF THE LUNGS, THROAT, AND NASAL PASSAGES, BY INHALATIONS OF ATOMIZED FLUIDS.

By Wm. C. Lyman, M.D., late Surgeon U. S. Navy, Resident Physician U. S. Marine Hospital, etc.

The use of atomized liquids has within the past year occupied a prominent place in therapeutics, as applied to diseases affecting the respiratory passages. The great number of cases of that class that occur in the higher latitudes, and their importance, as bearing upon the health, and often the life of the individual, render their correct treatment, and the use of every proper remedial measure, one of the most sacred duties of the physician. Although the use of atomized liquids has its opponents, as being an ineffectual measure, the profession not only has adopted it, but has found it a valuable adjunct to other means before practiced. It is to contribute to the sum of evidence upon this subject that I have been induced to make a series of observations, which form a part of this communication.

It is a fact, patent to every one, that the function of respiration plays a very important part in the human economy, and that poisonous gases and other substances are readily received into the body, and rapidly produce their characteristic, or specific, effects when introduced. It may be safely concluded that attempts to gain a beneficial influence through this function would not be abortive, for similar reasons.

It is also true that local medicaments have as important a place in therapeutics as those which are introduced into the system; in fact, there are many diseases that are now treated wholly by local applications that were formerly thought only to yield to mysterious combinations of drugs, which, by some power, neither known nor explained, accomplished the desired work: thus fostering that benighted condition that leads the Indian to implicitly believe his "medicine man," when, after some infernal incantation, he tells him some specious untruth, or that which is only another phase of the same love of the mysterious, or belief in that only which cannot be understood, by investing a simple drop of water, or grain of sugar, with a power that mocks the infinite.

Inhalations of atomized or pulverized fluids may be made available for the application of both local and general remedies, producing first their local effect, and, by absorption into the circulation, their habitual impressions. It is always well to produce as direct an impression as possible, thus requiring a less dose, and a less prolonged impression, as, for instance, the hyperdermic use of morphia, compared with its introduction by way of the stomach.

Inhalation as a remedy is no new thing, as every one knows who is at all familiar with the literature of the profession; but until the pulverization of fluids was accomplished and put in practical use, we were not one step in advance of the physicians of ancient Greece in this particular thing of inhalations But now we claim a step forward, that this is a valuable addition to therapeutics, as necessary as any other.

The various instruments and their parts have been so often and fully described in medical publications that I will only say that those made by Gemrig, of Philadelphia, and Codman & Shurtleff, of Boston, answer every purpose that can possibly be required of a steam instrument of this kind, and the one of Andrew Clark, which consists of two globes, to maintain a steady current of air, will, equally well, accomplish the object for which it is designed. A third instrument is that of Oliver's, which has the tube fitted to a glass receiver, containing the atomizing tube, and which has an opening for the mouth to receive the spray.

The method of administering these inhalations deserves, at least, a passing notice, for it is sometimes difficult to prove that the spray passes into the respiratory passages at all, but is claimed that it finds a lodgment upon the pharynx and glottis, the respired air then being deprived of its particles of fluid. But we find irrefragable proof that atoms of liquid thus produced, do penetrate into the minor branches, in the interesting experiments detailed before the Academy of Medicine in Paris, by M. Demarquay. One of these is thus described: "Several small animals, as rabbits, cats, and Guinea pigs, were forced to inspire an atomized solution of one of the per-salts of iron, for a period of nearly ten minutes, the nostrils being closed. The presence of iron, even in the pulmonary tissue itself, was plainly demonstrable by the proper tests, the prussiate of potash, and acetic acid. Also, a patient in the hospital, who had an opening in the trachea submitted to an experiment being made upon himself in the following manner: A probe, covered with paper moistened with tr. chlor. ferri, was introduced into the trachea, and a strong solution of tannic acid was inhaled for a period of three minutes, a decided black, inky color was obtained." It is a well established fact that coal miners, often present evidence of finely divided substances having found their way into the air vesicles themselves. We have often noticed in using either very warm or very cold vapors, that the sensation of warmth or of cold was experienced throughout the entire extent of both lungs.

While it must be acknowledged that atomized fluids find their way into the more minute bronchi and air vesicles themselves, it is impossible to say how great a part of every ounce of liquid thus used pervades, in this manner, the whole bronchial tract, and we can only approximate it by judging from the effect produced upon the individual. A œdema about the glottis, or swelling of the vocal cords, are obstacles of a mechanical nature, to the free ingress of vapor, and in any case of failure to obtain the desired effect, such obstacle should be sought for, and, if possible, removed. It is difficult to breathe the atomized fluid through the circuitous nasal passages, without raising the head, and taking care that the tongue is well depressed, and the body inclined so as to make the most favorable angles within the passage traversed by the vapor. The impinging of the current upon any salient point causes a precipitation that soon deprives the air of nearly every perceptible particle of vapor, and it passes into the lungs in its unmedicated condition. I have several times tested this matter, watching the inhalations closely, and examining the patient with the laryngoscope subsequently, using liquids of unmistakable color, and find that none had penetrated further than the fauces. Afterwards, when the patient had carefully adjusted his position, the color extended beyond the reach of the laryngoscope, and expectoration was tinged with the color used in the experiment, for some time after.

The application of remedies is the most important part of this subject that occupied our attention after demonstrating the possibility of their use in this manner. The observations of the writer extend over a series of about seventy cases of disease of the respiratory mucous membrane. It is impossible to give more than an outline of the treatment of these cases in an article of this length, embodying the general considerations only. About one-fourth of these cases were catarrhal inflammation of the nasal passages, in several cases extending to the pharynx. There was a profuse discharge from the nostrils—

sometimes of offensive character, occasionally streaked with blood, with pains about the frontal sinus, and an apparently thickened or swollen condition of the pituitary membrane. This condition had gradually come on through several months. There was a partial loss of smell, and the voice was considerably changed; there was found to be gradual and steady improvement under the use of a solution of chlorate of potash, alternated sometimes with sulphate of iron and tr. sanguinaria. The inhalations were rarely given more than twice daily. The chlorate of potash appears to be applicable to acute and subacute conditions, controlling inflammation and relieving discomfort in a remarkable degree. Acute conditions frequently require anodyne applications along with the chlorate, such as hyoscyamia, aqueous ext. of opium, or conium, to get the best result.

Some ten or twelve cases of pharyngitis, not all identical in character, came under observation—some of which were type cases of "clergyman's sore throat." These yielded readily to a solution of nitrate of silver, at first, and, subsequently, a strong solution of tannin. At the end of a week a marked change for the better was apparent. In one case, particularly, where the whole lining of the throat was intensely red and inflamed, wherever it could be brought into view by depressing the tongue, and by the use of the laryngoscope, from the palate to the glottis. The pulverized solution of nitrate of silver was used twice only before a great amelioration took place in all symptoms, checking the inflammation almost immediately. The spray is much preferable to sponging, as a more agreeable operation to the patient, and applies the agent to all parts—a difficult thing to do with a sponge, which only touches the most exposed portions, leaving those deeper more difficult, and often more inflamed parts, untouched by the remedy.

In syphilitic inflammations of the throat, great benefit followed the use of dilute preparations of creasote, corrosive sublimate, iodine, and permanganate of potash, but it is wholly improbable that these alone would have effected a cure, constitutional remedies being, of course, a *sine qua non.* Eleven cases of secondary syphilitic disease thus came under observation.

The remainder of the cases, included among those who came under particular observation, were of chronic bronchitis, some having tubercular deposit in the lungs, as determined by physical exploration of the chest, together with a profuse expectoration and cough. Some of the more advanced were only benefited as far as temporary relief of the cough and profuse expectoration. Others, less far advanced, decidedly improved, and gained flesh and strength, using cod liver oil, and other kindred remedies, also. Solutions of sulphate of copper, tannin, alum, acetate of lead, nitrate of silver, and Lugol's solution of iodine, with and without opium, were used in these cases. The preference is given to sulphate of copper, tannin, and nitrate of silver. I have only one case of hæmoptysis that was sufficiently well marked to be of importance. It occurred without much pain or febrile movement, in this instance; used the solution of per-sulphate of iron, much vaunted for its styptic properties; a very weak solution was inhaled with great good effect in promptly checking the hemorrhage. Opportunity occurred for the use of the atomizer, in a single case only, of whooping cough, occurring in a child of four years. Inhalations of dilute solution of belladonna, with occasional alternations, with solution of alum and conium, apparently greatly modified the severity of the paroxysms, they being less frequent and distressing, and of less duration, with easier expectoration.

A medical gentleman of this city informs me that he has used inhalations of lime water in two cases of inflammatory croup, in one of which preparation was being made for tracheotomy; recovery followed in both cases. The use of aqueous vapor is no new thing, it is true, but the prompt action leads to the opinion that the solvent power that the lime water exerted upon the inflammatory exudation within the trachea determined the favorable result.

The same remedy has become a popular one for the diptheritic exudations about the throat, and in the nostrils, and deservedly so, judging from the evidence upon this point contained in the late contributions to medical literature upon this subject. It is certainly the most available method of using any topical remedy.

When, on account of the tenderness and swelling, the application of the sponge is wholly impossible, and the use of a syringe wholly inadequate, we may consider it a very valuable addition to the remedial measures for these affections. The following cases, given in detail, will probably sufficiently illustrate the beneficial effects of this plan of treatment.

CASE I. J. W., native of the United States; had served three years in the army of the South-west, during which period he contracted a troublesome cough, which had existed for eighteen months, when he came under observation. He had been under treatment for months, going from one place and physician to another, but had probably been impatient, and failed to fully carry out any plan. He said he had a "*bushel of empty bottles at home*," showing that he had not been wanting in medicine, at least. He was emaciated, expectorating copiously; respiration at least 28 per minute; pulse about 90. He had little appetite; walked up a flight of stairs with great difficulty; coughed very much on lying down; complained of great pain in his chest, at times, and always when coughing. The alvine and urinary evacuations were normal in character. The most minute and repeated examinations failed to discover any tubercular deposit in the lungs, although expected; he had never had hæmoptysis. Failing to discover tuberculosis, it was concluded that there was only a general sub-acute bronchitis. He inhaled twice daily a solution of chlorate potass, with tr. opium, sometimes alternatated with acetate of lead and morphia; tr. sanguinaria was used at times, with each of the above preparations. He was given an expectorant mixture, of very common place character, more as a placebo, than anything else. He steadily improved from about the second day, when the beneficial effect of the remedy became obvious. The second night after beginning the inhalations was the first quiet night he had had for weeks; he expressed himself as very much relived, and very grateful withal. His appetite gradually returned; he gained weight; his respiration returned to a natural frequency. At the end of two weeks he could take five times the exercise that was possible at first; he coughed and expectorated comparatively little. When first used the vapor produced a suffocative feeling, with an almost incessant, short, hacking cough, which passed away in half an hour. As he become more accustomed to them, the inhalations were a source of comfort, producing no cough or dyspnœa. These were continued for about a month; the patient steadily improving, when he went to work, with only a trace of his cough left.

Case II. E. B., merchant, age about 45; has had catarrhal inflammation of the nasal passages for the past five years. It was originally a "cold," that, neglected and aggravated by exposure, has continued on till he finds himself in a condition that he describes himself, in nearly the following words: After recovering from the first symptoms of the cold I felt well, and thought I was well, but a slight discharge from the nostrils ensued, and slight soreness, which has always remained, growing worse and worse, with each slight exposure, till now I have constantly a good deal of discharge, often streaked with blood, and purulent in character, and of disagreeable odor. I cannot go to church, or to any place of amusement without being conscious that a disagreeable coughing, hawking, and expectoration may be unpleasant to those near me. The perception of odors is much less acute than formerly; am depressed; have lost between fifteen and twenty pounds in weight, and am really discouraged, having tried a great many medicines, and feel no permanent benefit from their use. This is, perhaps, a type of many cases that are be seen of inflammation of the nasal mucous membrane. The truth is that they are benefited in the same way by local treatment, that inflammation of the conjunctiva, and other mucous surfaces are. Injections with a syringe, and the use of powders, never more than partially accomplishes the desired object, reaching only a small part of the affected surfaces; whereas, the use of pulverized liquids enables us to apply the medicament to the whole. In this case the treatment was mainly the inhalation, twice daily, of an atomized solution of nitrate of silver, great care being used to prevent the discolorization of the face, by protecting the adjacent parts by covering with oil, and enveloping the face in a towel during the process of inhalation. After the first eight or ten days a solution of chlorate of potash was substituted, which was continued, with occasional alternations, with the nitrate, for several weeks. The progress toward recovery was regularly progressive; the mucous discharge becoming less in quantity; the offensive odor being controlled; the sense of smell reëstablished, and the patient improving in general condition. Chalybeates were prescribed in connection with the local means; but it is wholly improbable that they would have produced this effect alone. This patient was under observation for over two months.

Case III. R. G., native of Germany; age 42; has been a sailor for twenty years; has always been in robust health, notwithstanding the exposure incident to a sailor's life, till eight months ago, when he contracted a pneumonia, which left him with a cough, and greatly debilitated; he continued in this condition for weeks, notwithstanding a tonic course of treatment being pursued. The frothy and copious expectoration continued; slight dullness was observed under the right clavicle on percussion, and large, dry crepitus was developed, with prolonged expiration and bronchial voice. These, with the general indications, showed conclusively the presence of commencing tubercular infiltration. At this time a slight, passive hæmoptysis occurred, an ounce or so of nearly pure blood being expectorated. Several successive attacks followed, a few days apart, with the sputa usually streaked with blood during the interim. Inhalations of styptic character were used at this period, (Monsil's salt,) once daily, which arrested the hæmorrhage at once, and appeared to prevent its recurrence as before, the patient acquiring a great degree of relief from his cough and expectoration, and improving in general condition. How far the curative power of this measure extends as a remedy in tuberculosis, may possibly be determined by subsequent observations, at present, we know it is a valuable and convenient means of controlling an alarming symptom, which alone would entitle it to a favorable consideration.

The doses in which these preparations are to be used, constitute a point of practical importance, to meet which will be appended the following tabular statement. This statement is for adults, and is to be diminished for children, according to the usual rules, proportioning the dose for the age. Several tables of like character have been made public, and in general features they all necessarily correspond; but it is my desire to present the conclusions that whatever opportunity of observation and experience I have had, has enabled me to arrive at this calculation, shows the proper quantity to the oz. of water:

| | | |
|---|---|---|
| 1. | Tinct. ferri susqui chloridi, | 10 to 20 m. |
| 2. | Liq. ferri per-sulphatis, | 5 to 15 m. |
| 3. | Alum, | 10 to 20 grs. |
| 4. | Tannin, | 5 to 30 grs. |
| 5. | Sulph. zinc, | 2 to 5 grs. |
| 6. | Sulph. cupri, | 1 to 4 grs. |
| 7. | Argenti nitras, | 1 to 10 grs. |
| 8. | Potass. chloras, | 10 to 30 grs. |
| 9. | Lime water, | Officinal strength. |
| 10. | Opium ext., | $\frac{1}{8}$ to 1 gr. |
| 11. | Morphia, | $\frac{1}{16}$ to $\frac{1}{4}$ gr. |
| 12. | Liq. iodini comp., | 2 to 4 m. |

